# Association between thyroid function and acute mountain sickness upon rapid ascent to 3650 m in euthyroid lowlanders in China

**DOI:** 10.1113/EP092400

**Published:** 2025-05-05

**Authors:** Cencen Wu, Yuanyuan Fan, Jiageng Cai, Zhulan Cai, Qi Yu, Jiayu Li, Yuan Xu, Penghui Zhao, Yuwen Ruan, Yongqi Zhao, Jicheng Gong, Yifan Xu, Tong Zhu, Lingyun Zu

**Affiliations:** ^1^ Department of Cardiology and Institute of Vascular Medicine Peking University Third Hospital Beijing China; ^2^ State Key Laboratory of Vascular Homeostasis and Remodeling Peking University Beijing China; ^3^ NHC Key Laboratory of Cardiovascular Molecular Biology and Regulatory Peptides Peking University Beijing China; ^4^ Beijing Key Laboratory of Cardiovascular Receptors Research Beijing China; ^5^ Department of Cognitive and Stress Medicine Beijing Institute of Basic Medical Sciences Beijing China; ^6^ SKL‐ESPC & SEPKL‐AERM, College of Environmental Sciences and Engineering, and Centre for Environment and Health Peking University Beijing China

**Keywords:** acute mountain sickness, high altitude, thyroid hormone sensitivity, thyroid hormones

## Abstract

Acute mountain sickness (AMS) is a major health issue for lowlanders when they ascend rapidly to altitudes above 2500 m. Thyroid hormones are essential for adaptive responses to the hypoxic environment of high altitude. However, there is limited evidence regarding the association between thyroid function and the prevalence of AMS. This study included 70 healthy euthyroid Chinese lowlanders who ascended from Beijing (44 m above sea level) to Lhasa (3650 m above sea level) by aircraft (flight time, approximately 5 h). The dataset was divided into a training set (80%) and a validation set (20%) for the development and validation of a prediction model. Thyroid hormones, demographic data and blood biochemical data were measured in the week before the ascent. The subjects in the training set were divided into an AMS group and a non‐AMS group based on the 2018 Lake Louise Acute Mountain Sickness Scale score. Thyroid hormones and thyroid hormone sensitivity indices were compared between the groups. Multivariable logistic regression analysis revealed that a higher baseline total triiodothyronine (TT3) level (odds ratio (OR) 2.474, 95% confidence interval (CI) 1.083–5.653) and a higher free triiodothyronine/free thyroxine (FT3/FT4) ratio (OR 3.427; 95% CI 1.266–9.280) were independent risk factors for development of AMS. The receiver‐operating characteristic and calibration curves showed that the model had good predictive ability and consistency in both the training and validation sets. In China, euthyroid lowlanders with a higher TT3 level or FT3/FT4 ratio are more susceptible to AMS after exposure to high altitudes.

## Abstract (highlights)

- **What is the central question of this study?**
  Acute mountain sickness is a major health concern for individuals ascending rapidly to altitudes above 2500 m and thyroid hormones are essential for adaptive responses to high altitudes: is there an association between thyroid hormones and the prevalence of AMS
- **What is the main finding and its importance?**
  Individuals with a higher total triiodothyronine level or free triiodothyronine/free thyroxine ratio at low altitudes were more susceptible to AMS upon exposure to an altitude of 3650 m. This finding indicates that thyroid hormone levels may assist in identifying individuals who are susceptible to AMS.

## Graphical abstract

This study evaluated the association between baseline thyroid hormones levels, sensitivity index and the incidence of AMS in 70 healthy individuals traveling from Beijing (44 m above sea level) to Lhasa (3650 m above sea level). The dataset was divided into a training set (n = 56) and a validation set (n = 14) at an ratio of 8:2. Prediction model for AMS was developed using the training set and subsequently validated using the validation set.

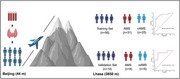

## 1 INTRODUCTION

A rapid ascent to high altitudes can lead to AMS, the main symptoms of which are headache, fatigue, dizziness, poor appetite, nausea and sleep disturbance. These symptoms typically manifest within 24 h of arrival at high altitudes but may appear as late as the third day (Basnyat & Murdoch, 2003; Gatterer et al., 2024; Hackett & Roach, 2001). AMS is usually benign and self‐limiting. However, approximately 1% of persons with AMS develop life‐threatening complications, such as high‐altitude pulmonary oedema or high‐altitude cerebral oedema (Imray et al., 2010; Luks et al., 2024; Meier et al., 2017). The prevalence of AMS varies according to acclimatization status, the absolute altitude reached, the rate of ascent and individual susceptibility (Basnyat & Murdoch, 2003; Gatterer et al., 2024). Unacclimatized individuals are at risk of AMS when they ascend to altitudes above 2500 m. However, previous studies and clinical experience suggest that susceptible individuals can develop AMS at as low as 2000 m (Honigman et al., 1993; Montgomery & Mills, 1989), and identification of those who are at increased risk of AMS before ascent to high‐altitude regions remains a formidable challenge.

Upon exposure to high altitude, hypoxia induces a physiological response to maintain adequate tissue oxygenation. However, an exaggerated or inappropriate response may have adverse effects leading to AMS (Gatterer et al., 2024). Several studies have identified severe hypoxaemia, sympathetic overactivity, increased salt and water retention, oxidative stress, and inflammation to be potential contributors to AMS (Bartsch & Swenson, 2013; Basnyat & Murdoch, 2003; Guo et al., 2024; Li et al., 2018; Meier et al., 2017). Acclimatization to a hypoxic environment is partially mediated by the balance of the sympathetic and parasympathetic nervous systems, and sympathetic overactivity may result in AMS (Imray et al., 2010; Simpson et al., 2019). Studies in trekkers have demonstrated that a shift toward sympathetic dominance at lower altitudes could be an early predictor of AMS at higher altitudes (Hamm et al., 2020; Huang et al., 2010).

Thyroid hormones play an essential role in the regulation of the adaptive stress response, energy metabolism and regulation of oxygen consumption, which are essential for acclimatization to the hypoxic environment of high altitude (Ma et al., 2004; Mullur et al., 2014; Richalet et al., 2010). Previous studies have consistently demonstrated that T3 and T4 increase independent of pituitary secretion of thyroid‐stimulating hormone (TSH) in response to exposure to high altitude (Keenan et al., 2020; Richalet et al., 2010; von Wolff et al., 2018), but the mechanism involved is still unclear. Considering that the thyroid gland is innervated by sympathetic branches, some researchers have speculated that sympathetic activation may be the cause of increased thyroid hormone levels at high altitudes (Richalet et al., 2010; Surks et al., 1967). Furthermore, hypoxia might directly promote the increased rate of release of thyroid hormones (Richalet et al., 2010).

The increase in thyroid hormone levels at high altitudes can increase cardiac output by increasing heart rate and cardiac contractility, induce production of erythrocytes via increased production of erythropoietin and hypoxia‐inducible factor 1, and shift the oxygen dissociation curve to the right, facilitating release of oxygen to the tissues under hypoxic conditions, which improves hypoxaemia and promotes acclimatization to high altitude (Ma et al., 2004; Sawhney & Malhotra, 1991; Snyder & Reddy, 1970; Szczepanek‐Parulska et al., 2017). However, the excessively increased thyroid hormone levels increase the oxygen demand in response to the increased basal metabolic rate, heart rate and thermogenesis, which may result in mal‐acclimatization under a hypoxic environment and trigger the development of AMS (Klein & Ojamaa, 2001; Mullur et al., 2014).

Previous studies have demonstrated that the increase in thyroid hormone levels at high altitudes is accompanied by an increase in the basal metabolic rate (Moncloa et al., 1966; Surks et al., 1967). We have speculated that individuals with higher baseline thyroid hormone levels at low altitudes may be more responsive to hypoxia and experience a further increase in these levels after exposure to high altitudes. Therefore, we hypothesized that the baseline thyroid hormone levels at low altitudes may serve as biomarkers of susceptibility to AMS.

In this study, we investigated the association of thyroid hormones and thyroid hormone sensitivity indices with AMS upon rapid ascent to high altitude in a cohort of euthyroid lowlanders who participated in the Physiological Adaptability after Short‐Term Exposure to Tibet Plateau trial (PASTE).

## 2 METHODS

### 2.1 Ethical approval

The study is registered with the Chinese Clinical Trial Registry (ChiCTR2000033451). The study protocol was approved by the Research Ethics Committee of Peking University Third Hospital (M2019452) and conducted in accordance with the principles of the *Declaration of Helsinki*. All participants provided written informed consent.

### 2.2 Experimental design and participants

**FIGURE 1:**
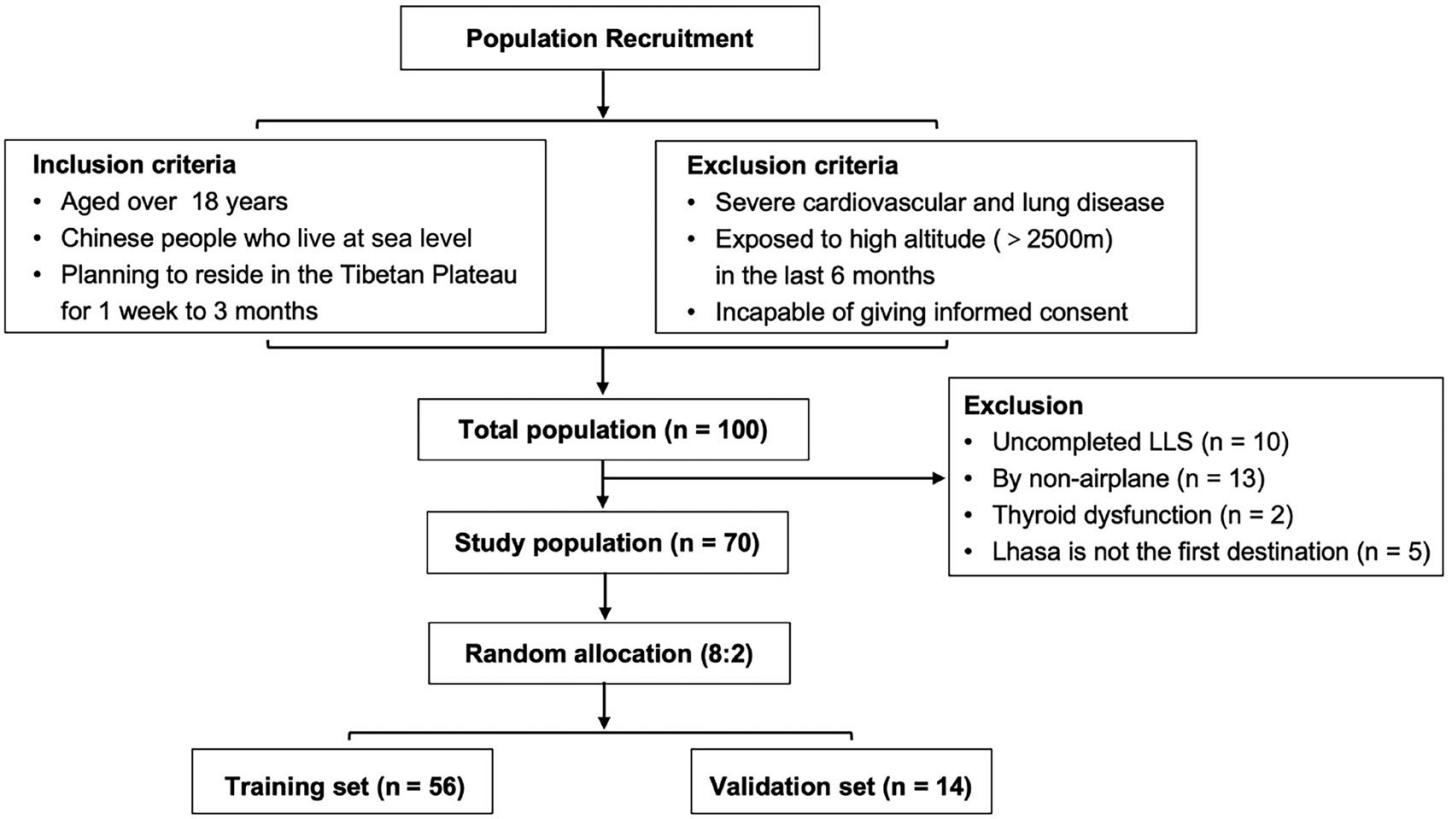
Flow chart showing the process used to select the study population. LLS, 2018 Lake Louise Acute Mountain Sickness Scale.

PASTE, as part of the Second Tibetan Plateau Scientific Expedition and Research Program, was a self‐comparison prospective cohort study launched by the Chinese Academy of Sciences and the Peking University Third Hospital and conducted between November 2019 and June 2024. One hundred healthy volunteers who were aged over 18 years and free from known cardiovascular, lung and other life‐threatening diseases were included. All participants were native lowlanders who had not been exposed to high altitude (>2500 m) for at least 6 months before participation in the study. Participants in PASTE remained continuously on the Tibetan Plateau (3100–6000 m above sea level) for 1 week to 3 months and underwent a series of health examinations, including laboratory investigations, an electrocardiogram, an echocardiogram and cardiopulmonary exercise testing within 1 week before departure from Beijing (44 m above sea level) and 1 week after leaving the Tibetan Plateau. We explored the association between baseline thyroid hormone levels recorded in Beijing and AMS during the first 3 days after arriving in the city of Lhasa (3650 m above sea level) by aircraft (flight time, approximately 5 h) using the data from PASTE. Participants with thyroid disease (*n* = 2), those with missing AMS‐related data (*n* = 10), those for whom the first destination was not Lhasa (*n* = 5), and those who did not arrive in Lhasa by aircraft (*n* = 13) were excluded. Finally, 70 individuals were included in the analysis. We randomly divided the dataset into training and validation sets in a ratio of 8 to 2, which has often been used in previous studies (Ahn et al., 2020; Jang et al., 2018; Liu et al., 2019). The model was constructed using the training set and validated in the validation set (Figure 1).

### 2.3 Anthropometric and biochemical measurements

Height and body weight were measured using standard methods. Body mass index (BMI) was calculated as body weight (kg) divided by the square of height (m). Systolic and diastolic blood pressures were measured twice using an electronic blood pressure monitor (HEM‐7430; Omron Corp., Tokyo, Japan) with the participant seated in a quiet and relaxed state. The average of two measurements was recorded. Venous blood samples were collected from participants between 08.00 and 10.00 h after fasting for 10 h within 1 week before departure to Lhasa from Beijing. The experimental procedure adhered strictly to the recommended guidelines for collection of blood samples by venipuncture (Institue, 2007) and the sample processing procedure (Institute, 2010). Total triiodothyronine (TT3), total thyroxine (TT4), free triiodothyronine (FT3), free thyroxine (FT4) and TSH levels were measured in serum using a chemiluminescence analyser (ADVIA Centaur XPT System; Siemens Healthineers, New York, USA). Blood counts were measured using an XN automated blood cell analyser (Sysmex, Kobe, Japan). Serum creatinine, total cholesterol, triglyceride, low‐density and high‐density lipoprotein cholesterol, alanine aminotransferase, aspartate aminotransferase, total protein, albumin and ultrasensitive C‐reactive protein levels were detected using an automatic biochemical analyser (Beckman Coulter, Brea, CA, USA).

### 2.4 Definition of acute mountain sickness

AMS was diagnosed using the 2018 Lake Louise Acute Mountain Sickness Scale (LLS) scoring system (Roach et al., 2018). The LLS score is the sum of the scores for four symptoms (headache, gastrointestinal symptoms, fatigue and/or weakness, and dizziness/light‐headedness). The score was calculated from the answers to a self‐reported electronic questionnaire that the study participants completed independently on the third day after arrival in Lhasa. We required the subjects to recall all AMS‐related symptoms that occurred during the first 3 days after their arrival in Lhasa. A total score of ≥3 points that includes at least one headache symptom is defined as AMS. A score of 3–5 points is defined as mild AMS, a score of 6–9 points as moderate AMS, and a score of 10–12 points as severe AMS.

### 2.5 Assessment of thyroid function

The normal laboratory reference intervals for TSH, TT4, TT3, FT4 and FT3 are 0.55–4.78 mIU/L, 4.50–10.90 µg/dL, 0.60–1.81 µg/L, 0.89–1.76 ng/dL (11.50–22.70 pmol/L) and 2.30–4.20 pg/mL (3.50–6.50 pmol/L), respectively. Consistent with previous research, euthyroidism was defined as TSH, TT4, TT3, FT4 and FT3 levels within the reference interval (Baumgartner et al., 2017; Cappola et al., 2006; Tseng et al., 2012; Wan et al., 2023).

The peripheral thyroid hormone sensitivity index was calculated as follows:

$$\mathrm{FT}3/\mathrm{FT}4\;\mathrm{ratio}=\mathrm{FT}3\;\left(\mathrm{pmol}/L\right)/\mathrm{FT}4\left(\mathrm{pmol}/L\right).$$

Higher values indicate higher peripheral sensitivity to thyroid hormones (Park et al., 2017). The central thyroid hormone sensitivity index includes the parametric thyroid feedback quantile‐based index (PTFQI), TSH index (TSHI), and thyrotrophin T4 resistance index (TT4RI). Negative PTFQI values indicate higher central sensitivity to a change in FT4, and positive values indicate lower central sensitivity. Higher TSHI and TT4RI values indicate decreased central sensitivity to thyroid hormones (Laclaustra et al., 2019). The PTFQI applies to various study populations. In this analysis, the μ FT4, δ FT4, μ lnTSH, and δ lnTSH values were 16.5335, 2.4729, 0.8781, and 0.4599, respectively (where μ represents the arithmetic mean of the distribution, and δ represents the standard deviation of the distribution). These values can be easily calculated using the following Excel spreadsheet formulae (Laclaustra et al., 2019):

$$\begin{array}{ccc} \mathrm{PTFQI} & = & \mathrm{NORM}.\mathrm{DIST}\;\left(\mathrm{FT}4,\;16.5335,\;2.4729,\;\mathrm{TRUE}\right)\\ & & +\mathrm{NORM}.\mathrm{DIST}\;(\mathrm{LN}\;\left(\mathrm{TSH}\right),0.8781,0.4599,\;\mathrm{TRUE})-1. \end{array}$$

$$\mathrm{TSHI}=\mathrm{Ln}\;\mathrm{TSH}\;\left(\mathrm{mIU}/\mathrm{mL}\right)+0.1345\times \mathrm{FT}4\;\left(\mathrm{pmol}/L\right).$$

$$\mathrm{TT}4\mathrm{RI}=\mathrm{FT}4\;\left(\mathrm{pmol}/L\right)\times \mathrm{TSH}\;\left(\mathrm{mIU}/\mathrm{mL}\right).$$

### 2.6 Sample size evaluation

The sample size was calculated using PASS (version 21.0.3; NCSS LLC, Kaysville, UT, USA). A two‐group (positive/negative) design with discrete response data was used to test the area under the receiver‐operating characteristic (ROC) curve against the null value of 0.5. The comparison was made using a two‐sided *Z*‐test with a type I error rate (α) of 0.05. The area under the curve (AUC) was computed between the *x*‐axis values of 0 and 1. The ratio of the standard deviation of the responses in the negative group to the standard deviation of the responses in the positive group was assumed to be 1. To detect an AUC of 0.75 with 90% power, 26 subjects were needed in the positive group and 26 in the negative group. A total of 52 subjects were required.

### 2.7 Statistical analysis

All statistical analyses were performed using IBM SPSS Statistics software (version 27; IBM Corp., Armonk, NY, USA) and R (version 4.4.0; R Foundation for Statistical Computing, Vienna, Austria). The study participants were randomly divided into the training set and validation set in a ratio of 8:2 with a fixed random seed to ensure the robustness and discrimination ability of the model.

The Kolmogorov–Smirnov test was used to test for normality of distributions after the subjects were divided into an AMS group and a non‐AMS group according to their LLS score. Continuous data that were normally distributed are shown as the mean ± standard deviation and those that had a skewed distribution as the median (interquartile range). Categorical data are shown as number (percentage). Differences in normally distributed variables were compared between the two groups using an unpaired Student's *t*‐test. Variables with a skewed distribution were compared between groups using the Mann–Whitney *U*‐test. The chi‐squared test was used as appropriate for between‐group comparisons of categorical variables.

In the training set, there were significant differences in the FT3/FT4 ratio and the TT3 level between the AMS and non‐AMS groups. Therefore, we standardized the FT3/FT4 ratio and the TT3 values using the *z*‐score and explored the relationship between the FT3/FT4 ratio, TT3 and AMS in a multiple logistic regression model after adjustment for age, sex and BMI, all of which may affect thyroid hormone levels. The variables included in the full model were used to construct the nomogram. The AUC for the ROC curve was used to validate discrimination efficiency, and the calibration curve was used to evaluate the calibration performance of the predictive model. Multiple corrections were performed using Bonferroni's method. A two‐sided *P*‐value of <0.05 was considered statistically significant.

## 3 RESULTS

### 3.1 Study population and baseline characteristics

The demographic and clinical characteristics of the study population are summarized in Table 1. Seventy subjects (median age 29 years, 42.9% male) were included. Thirty‐nine (55.7%) were diagnosed to have AMS based on their LLS score. AMS was mild in 28 study participants and moderate in 11. There were no cases of severe AMS. None of the subjects experienced high‐altitude pulmonary oedema or high‐altitude cerebral oedema. After random allocation, there were 56 subjects in the training set and 14 in the validation set. There was no significant difference in age, sex, BMI, systolic or diastolic blood pressure, heart rate, personnel type, prevalence of AMS, LLS total score, or blood biochemical indices between the training and validation sets.

**TABLE 1:** Demographic and clinical characteristics of the training and validation sets.

Characteristic	Total (*n* = 70)	Training set (*n* = 56)	Validation set (*n* = 14)	*P*
Demographics				
Age (years)	29.00 (24.75, 39.00)	29.00 (24.25, 38.75)	29.50 (25.50, 39.25)	0.664
Sex (*n* (%))				1.000
Male	30 (42.9)	24 (42.9)	6 (42.9)	
Female	40 (57.1)	32 (57.1)	8 (57.1)	
BMI (kg/m^2^)	22.88 ± 2.97	22.89 ± 3.07	22.84 ± 2.61	0.961
SBP (mmHg)	113 (106, 120)	113 (106, 120)	114 (105, 128)	0.746
DBP (mmHg)	67 (59, 73)	67 (59, 75)	67 (62, 72)	0.906
HR (bpm)	66 ± 8	66 ± 8	65 ± 8	0.677
Personnel type (*n* (%))				0.436
Scientific researcher	58 (82.9)	45 (80.4)	13 (92.9)	
Tourist	12 (17.1)	11 (19.6)	1 (7.7)	
LLS score				
AMS (*n* (%))	39 (55.7)	31 (55.4)	8 (57.1)	0.904
Total score	3.00 (1.00, 5.00)	3.50 (1.00, 5.00)	3.00 (1.75, 4.00)	0.806
Headache	1.00 (0.00, 2.00)	1.00 (0.00, 2.00)	1.00 (0.00, 1.00)	0.901
Gastrointestinal symptoms	0.00 (0.00, 1.00)	0.00 (0.00, 1.00)	0.00 (0.00, 1.00)	0.779
Fatigue	1.00 (0.00, 2.00)	1.00 (1.00, 2.00)	1.00 (1.00, 1.25)	0.955
Dizziness	1.00 (0.00, 1.00)	1.00 (0.00, 1.00)	1.00 (0.00, 1.00)	0.935
Laboratory values				
WBC (×10^9^/L)	5.66 ± 1.24	5.64 ± 1.27	5.73 ± 1.16	0.813
HGB (g/L)	142.44 ± 15.90	141.73 ± 15.60	145.29 ± 17.34	0.458
PLT (×10^9^/L)	253.87 ± 49.53	253.81 ± 50.69	254.14 ± 46.37	0.982
ALT (U/L)	16.50 (12.00, 25.00)	18.50 (12.25, 27.50)	14.50 (8.00, 16.25)	0.034
AST (U/L)	21.00 (18.00, 24.00)	21.00 (18.25, 24.00)	19.50 (17.00, 21.50)	0.185
TP (g/L)	76.15 ± 4.38	76.17 ± 4.45	76.07 ± 4.26	0.941
ALB (g/L)	47.28 ± 2.82	47.57 ± 2.87	46.13 ± 2.34	0.087
BUN (mmol/L)	4.73 ± 1.01	4.69 ± 1.02	4.90 ± 0.98	0.481
Cr (mmol/L)	75.07 ± 12.20	75.04 ± 11.84	75.21 ± 14.05	0.961
UA (µmol/L)	346.31 ± 80.28	342.48 ± 82.13	361.64 ± 73.16	0.428
TC (mmol/L)	4.78 ± 0.85	4.79 ± 0.85	4.72 ± 0.86	0.772
HDL‐C (mmol/L)	1.36 (1.12, 1.58)	1.38 (1.17, 1.59)	1.32 (1.10, 1.51)	0.366
LDL‐C (mmol/L)	2.76 (2.25, 3.13)	2.76 (2.31, 3.11)	2.80 (2.05, 3.24)	0.982
USCRP (mg/L)	0.46 (0.24, 1.23)	0.44 (0.24, 1.32)	0.60 (0.16, 1.01)	0.993
HbA1c (%)	5.40 (5.10, 5.53)	5.40 (5.13, 5.60)	5.40 (5.08, 5.50)	0.652
TT3 (µg/L)	1.09 ± 0.19	1.09 ± 0.20	1.05 ± 0.17	0.480
TT4 (µg/dL)	7.34 ± 1.65	7.37 ± 1.77	7.24 ± 1.06	0.805
FT3 (pmol/L)	5.19 ± 0.72	5.19 ± 0.72	5.21 ± 0.75	0.928
FT4 (pmol/L)	16.38 (14.66, 17.81)	16.38 (14.37, 17.78)	16.38 (15.44, 17.94)	0.386
TSH (mIU/L)	2.15 (1.69, 3.10)	2.15 (1.69, 2.98)	2.31 (1.70, 3.82)	0.638
FT3/FT4 ratio	0.32 ± 0.04	0.32 ± 0.04	0.31 ± 0.05	0.238
TSHI	3.08 ± 0.54	3.04 ± 0.55	3.24 ± 0.51	0.207
TT4RI	36.84 (27.02, 54.32)	36.63 (26.42, 52.72)	43.15 (27.20, 62.27)	0.347
PTFQI	0.92 ± 0.38	−0.07 ± 0.39	0.05 ± 0.33	0.281

*Note*: Continuous variables are presented as the mean ± standard deviation or as the median (interquartile range). Subtype variables are presented as the frequency (percentage). Abbreviations: ALB, albumin; ALT, alanine aminotransferase; AMS, acute mountain sickness; AST, aspartate aminotransferase; BMI, body mass index; BUN, blood urea nitrogen; Cr, creatinine; DBP, diastolic blood pressure; FT3, free triiodothyronine; FT3/FT4 ratio, free triiodothyronine to free thyroxine ratio; FT4, free thyroxine; HbA1c, glycated haemoglobin; HDL‐C, high‐density lipoprotein cholesterol; HGB, haemoglobin level; HR, heart rate; LDL‐C, low‐density lipoprotein cholesterol; LLS, 2018 Lake Louise Acute Mountain Sickness Scale; PLT, platelet count; PTFQI, parametric thyroid feedback quantile‐based index; SBP, systolic blood pressure; TC, total cholesterol; TP, total protein; TSH, thyroid‐stimulating hormone; TSHI, thyroid‐stimulating hormone index; TT3, total triiodothyronine; TT4, total thyroxine; TT4RI, thyrotrophin thyroxine resistance index; UA, uric acid; USCRP, ultrasensitive C‐reactive protein; WBC, white blood cell count.

### 3.2 Baseline characteristics in the training set

In the training set, there was no significant difference in age, sex, BMI, systolic or diastolic blood pressure, heart rate, or personnel type between the AMS and non‐AMS groups. Laboratory tests indicated that the white blood cell count and haemoglobin, platelet count, aspartate aminotransferase, alanine aminotransferase, creatinine, blood urea nitrogen, and lipid profiles were similar between the two groups (Table 2). The thyroid hormone levels were also similar between the groups, except for TT3, which was significantly higher in the AMS group (1.16 ± 0.17 pmol/L vs. 1.01 ± 0.19 pmol/L; *P* = 0.005). There were no significant between‐group differences in the thyroid hormone sensitivity indices, except for a considerably higher FT3/FT4 ratio in the AMS group (0.34 ± 0.04 vs. 0.30 ± 0.03; *P *< 0.001).

**TABLE 2:** Demographic and clinical characteristics of the AMS and non‐AMS groups in the training set.

Characteristic	Total (*n* = 56)	AMS group (*n* = 31)	Non‐AMS group (*n* = 25)	*P*
Demographics				
Age (years)	29.00 (24.25, 38.75)	27.00 (24.00, 38.00)	33.00 (25.00, 49.00)	0.418
Sex (*n* (%))				0.214
Male	24 (42.9)	11 (35.5)	13 (52.0)	
Female	32 (57.1)	20 (64.5)	12 (48.0)	
BMI (kg/m^2^)	22.89 ± 3.07	22.73 ± 2.90	23.09 ± 3.32	0.664
SBP (mmHg)	113 (106, 120)	113 (106, 116)	114 (107, 123)	0.729
DBP (mmHg)	67 (59, 75)	67 (59, 68)	68 (57, 79)	0.342
HR (bpm)	66 ± 8	66 ± 9	67 ± 7	0.828
Personnel type (*n* (%))				0.514
Scientific researcher	45 (80.4)	26 (83.9)	19 (76.0)	
Tourist	11 (19.6)	5 (16.1)	6 (24.0)	
LLS score				
Total score	3.50 (1.00, 5.00)	4.00 (4.00, 6.00)	1.00 (0.00, 2.00)	< 0.001
Headache	1.00 (0.00, 2.00)	2.00 (1.00, 2.00)	0.00 (0.00, 0.50)	< 0.001
Gastrointestinal symptoms	0.00 (0.00, 1.00)	1.00 (0.00, 1.00)	0.00 (0.00, 0.00)	< 0.001
Fatigue	1.00 (1.00, 2.00)	1.00 (1.00, 2.00)	1.00 (0.00, 1.00)	< 0.001
Dizziness	1.00 (0.00, 1.00)	1.00 (1.00, 1.00)	0.00 (0.00, 0.50)	< 0.001
Laboratory values				
WBC (×10^9^/L)	5.64 ± 1.27	5.66 ± 1.32	5.62 ± 1.22	0.891
HGB (g/L)	141.73 ± 15.60	141.74 ± 14.69	141.72 ± 16.98	0.996
PLT (×10^9^/L)	253.80 ± 50.69	261.29 ± 55.02	244.52 ± 44.08	0.222
ALT (U/L)	18.50 (12.25, 27.50)	17.00 (12.00, 28.00)	19.00 (13.00, 26.00)	0.662
AST (U/L)	21.00 (18.25, 24.00)	21.00 (17.00, 24.00)	22.00 (19.50, 25.00)	0.221
TP (g/L)	76.17 ± 4.45	76.35 ± 4.27	75.95 ± 4.74	0.746
ALB (g/L)	47.57 ± 2.87	47.56 ± 2.74	47.58 ± 3.09	0.981
BUN (mmol/L)	4.55 (3.83, 5.58)	4.40 (3.80, 5.60)	4.70 (4.10, 5.60)	0.494
Cr (mmol/L)	75.04 ± 11.84	73.74 ± 9.55	76.64 ± 14.22	0.367
UA (µmol/L)	342.48 ± 82.13	337.65 ± 92.06	348.48 ± 69.27	0.628
TC (mmol/L)	4.79 ± 0.85	4.73 ± 0.60	4.87 ± 1.09	0.571
HDL‐C (mmol/L)	1.41 ± 0.32	1.39 ± 0.26	1.43 ± 0.39	0.685
LDL‐C (mmol/L)	2.79 ± 0.69	2.77 ± 0.55	2.81 ± 0.86	0.832
USCRP (mg/L)	0.44 (0.24, 1.32)	0.44 (0.22, 1.48)	0.44 (0.24, 1.01)	0.508
HbA1c (%)	5.40 (5.13, 5.60)	5.40 (5.10, 5.60)	5.50 (5.30, 5.65)	0.077
TT3* (µg/L)	1.09 ± 0.20	1.16 ± 0.17	1.01 ± 0.19	0.005
TT4 (µg/dL)	7.37 ± 1.77	7.78 ± 1.29	6.85 ± 2.15	0.065
FT3 (pmol/L)	5.19 ± 0.72	5.30 ± 0.65	5.05 ± 0.79	0.197
FT4 (pmol/L)	16.25 ± 2.50	15.89 ± 2.25	16.70 ± 2.75	0.231
TSH (mIU/L)	2.15 (1.69, 2.98)	2.10 (1.69, 3.98)	2.25 (1.68, 2.83)	0.382
FT3/FT4 ratio*	0.32 ± 0.04	0.34 ± 0.04	0.30 ± 0.03	0.001
TSHI	3.04 ± 0.55	3.02 ± 0.57	3.06 ± 0.53	0.780
TT4RI	36.63 (26.42, 52.72)	36.58 (25.17, 64.90)	38.67 (28.39, 51.32)	0.252
PTFQI	−0.07 ± 0.40	−0.09 ± 0.41	−0.05 ± 0.38	0.727

*Note*: Continuous variables are presented as the mean ± standard deviation or as the median (interquartile range). Subtype variables are presented as the frequency (percentage). The asterisk (*) indicates a statistically significant significance between the AMS and non‐AMS groups in the training set after Bonferroni correction for multiple testing. Abbreviations: ALB, albumin; ALT, alanine aminotransferase; AMS, acute mountain sickness; AST, aspartate aminotransferase; BMI, body mass index; BUN, blood urea nitrogen; Cr, creatinine; DBP, diastolic blood pressure; FT3, free triiodothyronine; FT3/FT4 ratio, free triiodothyronine to free thyroxine ratio; FT4, free thyroxine; HbA1c, glycated haemoglobin; HDL‐C, high‐density lipoprotein cholesterol; HGB, haemoglobin level; HR, heart rate; LDL‐C, low‐density lipoprotein cholesterol; LLS, 2018 Lake Louise Acute Mountain Sickness Scale; PLT, platelet count; PTFQI, parametric thyroid feedback quantile‐based index; SBP, systolic blood pressure; TC, total cholesterol; TP, total protein; TSH, thyroid‐stimulating hormone; TSHI, thyroid‐stimulating hormone index; TT3, total triiodothyronine; TT4, total thyroxine; TT4RI, thyrotrophin thyroxine resistance index; UA, uric acid; USCRP, ultrasensitive C‐reactive protein; WBC, white blood cell count.

### 3.3 Construction of the prediction model in the training set

We constructed four models to investigate the association between thyroid function and AMS using TT3, the FT3/FT4 ratio, and other covariables, including sex, age and BMI. In Model 1 and Model 2, TT3 and the FT3/FT4 ratio were used as independent variables, and AUC values were 0.697 (95% CI 0.559–0.834) and 0.737 (95% CI 0.608–0.866), respectively. When both TT3 and the FT3/FT4 ratio were incorporated simultaneously in Model 3, the AUC increased to 0.786 (95% CI 0.667–0.905), indicating that the combination of these two variables enhanced the predictive capacity of the model. Model 4 further integrated sex, age and BMI as additional covariates, which may affect thyroid hormone levels, based on Model 3, resulting in a notable improvement in the AUC to 0.861 (0.758–0.963). These results suggested that TT3 and the FT3/FT4 ratio were key predictive factors, and the additional variables of sex, age and BMI significantly improved the discriminative power of the model. The baseline FT3/FT4 ratio (OR 3.427; 95% CI 1.266–9.280) and the TT3 level (OR 2.474; 95% CI 1.083–5.653) were identified to be independent predictors of susceptibility to AMS after exposure to high altitudes (Table 3).

**TABLE 3:** Results of binary logistic regression and receiver‐operating characteristic curve analyses for the four models in the training set.

Model	Variable	β	OR (95% CI)	AUC (95% CI)
1	TT3	0.811	2.251 (1.213–4.178)	0.697 (0.559–0.834)
2	FT3/FT4 ratio	1.166	3.209 (1.472–6.995)	0.737 (0.608–0.866)
3	TT3	0.642	1.901 (0.979–3.689)	0.786 (0.667–0.905)
	FT3/FT4 ratio	1.020	2.773 (1.235–6.227)	
4	TT3	0.906	2.474 (1.083–5.653)	0.861 (0.758–0.963)
	FT3/FT4 ratio	1.232	3.427 (1.266–9.280)	
	Sex			
	Female		Reference	
	Male	−0.893	0.410 (0.081–2.067)	
	Age	−0.060	0.942 (0.880–1.008)	
	BMI	−0.051	0.951 (0.763–1.184)	

*Note*: The FT3/FT4 ratio and TT3 levels were standardized using the *z*‐score transform method. The ORs for the FT3/FT4 ratio and TT3 indicated that a 1 standard deviation change resulted in a change of (OR − 1).

Abbreviations: AUC, area under curve; BMI, body mass index; CI, confidence interval; FT3/FT4 ratio, free triiodothyronine to free thyroxine ratio; OR, odds ratio; TT3, total triiodothyronine.

### 3.4 Development of a nomogram from the prediction model

**FIGURE 2:**
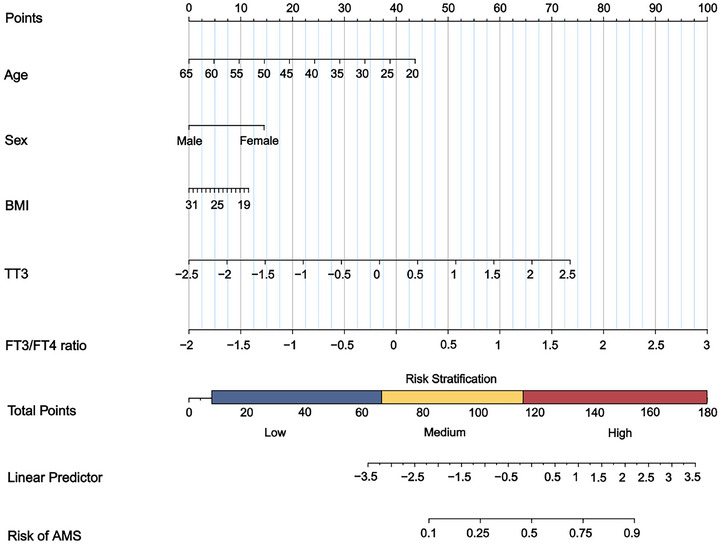
Nomogram for the training set used to predict AMS. AMS, acute mountain sickness; BMI, body mass index; FT3/FT4 ratio, free triiodothyronine to free thyroxine ratio; TT3, total triiodothyronine.

We illustrated model 4 in the form of a nomogram so that the contribution of each variable to the overall risk dimensions could be visualized. In the nomogram, the score assigned to each variable was proportional to its contribution to the risk of AMS. The total score on the risk axis represents the probability of AMS. The higher the score, the higher the risk of developing AMS after exposure to high altitudes (Figure 2).

### 3.5 Evaluation of model discrimination and calibration in the training set

**FIGURE 3:**
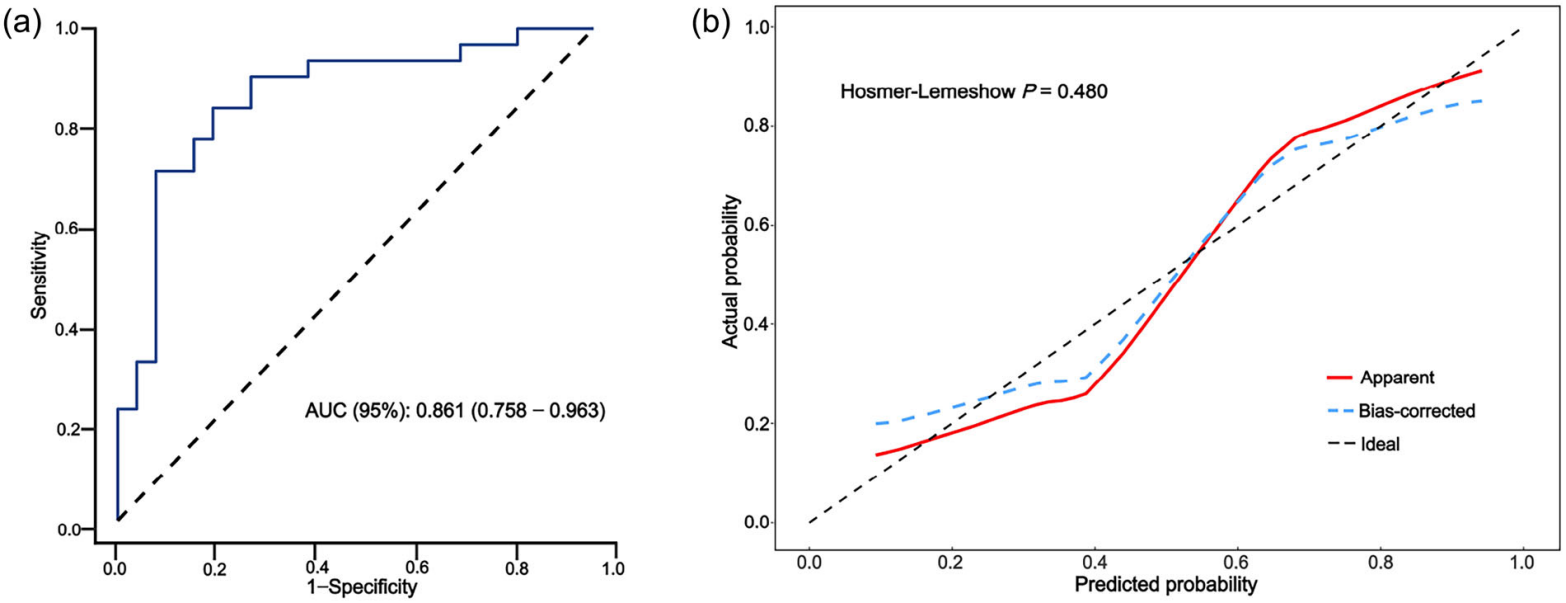
Receiver‐operating characteristic (a) and calibration (b) curves for Model 4 in the training set. AUC, area under the curve.

The ROC curve constructed to evaluate the ability of the model to predict AMS had an AUC of 0.861 (95% CI 0.758–0.963) in the training set, indicating that the model had good discriminatory performance with low error (Figure 3a). The calibration curve for the prediction performance of the model showed good consistency between the predicted and observed outcomes (Figure 3b).

### 3.6 Internal validation of the prediction model in the validation set

**FIGURE 4:**
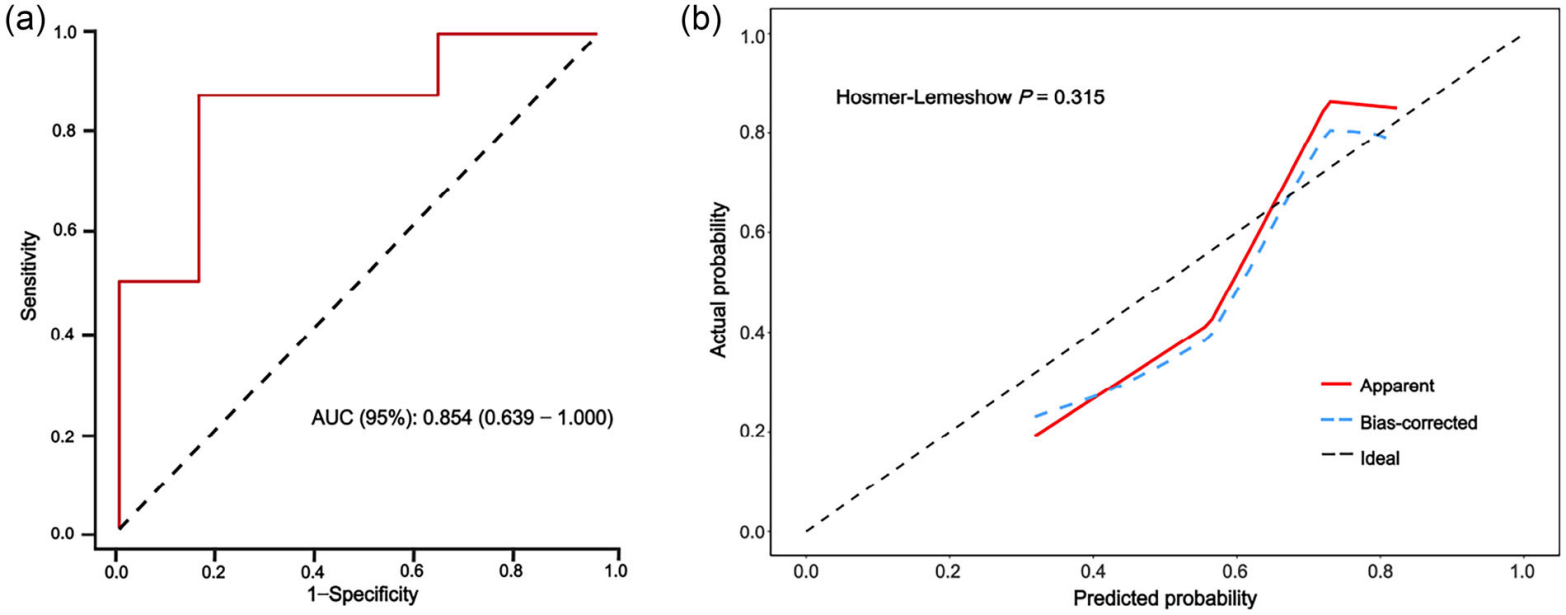
Receiver‐operating characteristic (a) and calibration (b) curves for Model 4 in the validation set. AUC, area under the curve.

In the validation set, the ROC curve constructed to evaluate the ability of the model to predict AMS had an AUC of 0.854 (95% CI 0.639–1.000), indicating good discriminatory performance with low error (Figure 4a). The calibration curve for the model showed good consistency between the predicted and observed outcomes (Figure 4b).

## 4 DISCUSSION

This study is the first to explore the relationship between thyroid function and the prevalence of AMS upon ascent to high altitude in euthyroid lowlanders. The main finding was that the baseline TT3 level and the FT3/FT4 ratio at low altitudes are independent predictors of susceptibility to AMS after exposure to high altitudes. Overall, our findings suggest that the TT3 level and FT3/FT4 ratio are valuable indices for the identification of individuals who are susceptible to AMS when they fly from a low altitude to a high altitude.

Thyroid hormones have been demonstrated to play an essential role in the regulation of human metabolism. Subtle changes in thyroid hormone levels, even within the normal range, can have a significant effect on the risk of metabolic diseases, including diabetes mellitus, hyperuricaemia, fatty liver, cardiovascular disease and even all‐cause mortality (Ding et al., 2022; Sun et al., 2022; Wan et al., 2024, 2023; Yu et al., 2024). Therefore, we believe that even if differences in thyroid hormone levels in euthyroid persons are minute, thyroid hormones can still serve as valuable biomarkers for the identification of individuals susceptible to AMS.

### 4.1 Possible mechanisms underlying the thyroid hormones response to hypoxia in AMS

Thyroid hormones are also key regulators of the stress response and energy balance and play an important role in acclimatization to high altitudes. Previous studies have consistently demonstrated increases in thyroid hormone levels in response to acute exposure to high altitudes, with increases in T3 and T4 but no change in the TSH level (Barnholt et al., 2006; Richalet et al., 2010; von Wolff et al., 2018). Several explanations have been proposed for the response of thyroid hormones to hypoxic acclimatization. An increase in circulating thyroid hormones markedly increases heart rate and myocardial contractility, which increases cardiac output to maintain an adequate oxygen supply (Klein & Ojamaa, 2001; Mullur et al., 2014). Furthermore, thyroid hormones promote production of erythrocytes and improve their oxygen‐carrying capacity (Szczepanek‐Parulska et al., 2017). Studies have found that thyroid hormones can stimulate the synthesis of 2,3‐diphosphoglycerate, which shifts the oxygen dissociation curve to the right, increasing oxygen delivery to tissues under hypoxic conditions (Sawhney & Malhotra, 1991; Snyder & Reddy, 1970). Therefore, increased thyroid hormone levels are regarded as a stress‐protective response that maintains sufficient tissue oxygenation and enables the body to acclimatize to a high‐altitude hypoxic environment. However, exaggerated or inappropriate responses may cause adverse effects and AMS (Gatterer et al., 2024). Inappropriately increased thyroid hormone levels in individuals with higher baseline levels of these hormones promote tachycardia, increased thermogenesis in tissues and higher resting metabolic expenditure, which further increases the tissue oxygen demand in a hypoxic environment (Klein & Ojamaa, 2001; Mullur et al., 2014). A decrease in oxygen supply and an imbalance in demand may exaggerate hypoxaemia and induce AMS. The findings of this study provide new insights into the mechanism of AMS. However, the current research on the response of thyroid hormones to hypoxia and its relationship with AMS is limited. More basic and clinical studies are needed to clarify the relationship between thyroid hormone levels and AMS.

### 4.2 Study limitations

This study has some limitations. First, it was performed at a single academic medical centre and lacks external validation, which may limit its representativeness and generalizability. Furthermore, its observational nature meant that it was not possible to determine whether there is a direct causal relationship between thyroid function and the likelihood of AMS. Second, although we controlled for many potential confounders, our findings may have been affected by factors such as ambient temperature, exercise and nutrition, which have also been associated with AMS (Guo et al., 2024; Khodaee et al., 2016). Third, 10% of our study population did not have an LLS score available and were excluded. However, there was no difference in baseline characteristics between the study population and the group that was excluded. Moreover, we required the subjects to complete the LLS questionnaire on the third day of the high‐altitude exposure and recall all the symptoms that occurred during the first three days after they arrived in Lhasa, which may lead to recalling bias. Finally, the study focused primarily on a healthy Chinese population and the sample size was relatively small. More basic and clinical studies, which dynamically monitor thyroid hormone changes at high altitudes are needed to clarify the relationship between thyroid hormone levels and AMS. Further studies are needed to demonstrate causality and determine the extent to which our findings are generalizable to other ethnicities and regions.

### 4.3 Conclusion

This study found that a higher TT3 level and a higher FT3/FT4 ratio are positively correlated with the development of AMS, indicating that people with a higher baseline TT3 and greater peripheral thyroid hormone sensitivity may be more susceptible to AMS. This finding may be important for early identification, prevention, and treatment of susceptible individuals. Larger prospective cohort studies are needed to confirm our findings.

## AUTHOR CONTRIBUTIONS

Conceptualization, data curation, formal analysis, and writing—original draft: Cencen Wu, Yuanyuan Fan. Formal analysis and visualization, writing—review and editing: Jiageng Cai, Zhulan Cai. Qi Yu. Data curation, writing—review and editing: Jiayu Li, Yuan Xu, Penghui Zhao, and Yuwen Ruan. Writing—review and editing: Yongqi Zhao, Jicheng Gong, and Yifan Xu. Conceptualization, writing—review and editing: Tong Zhu. Conceptualization, funding acquisition, writing—review and editing: Lingyun Zu. All authors approved the final version of the manuscript and agree to be accountable for all aspects of the work in ensuring that questions related to the accuracy or integrity of any part of the work are appropriately investigated and resolved. All persons designated as authors qualify for authorship, and all those who qualify for authorship are listed.

## CONFLICT OF INTEREST

None declared.

## Data Availability

The data that support the findings of this study are available from the corresponding author on reasonable request.
